# RNA Interference Mediated Inhibition of Dengue Virus Multiplication and Entry in HepG2 Cells

**DOI:** 10.1371/journal.pone.0034060

**Published:** 2012-03-23

**Authors:** Mohammed Abdelfatah Alhoot, Seok Mui Wang, Shamala Devi Sekaran

**Affiliations:** 1 Department of Medical Microbiology, Faculty of Medicine, University of Malaya, Kuala Lumpur, Malaysia; 2 Institute of Medical Molecular Biotechnology, Faculty of Medicine, Universiti Teknologi MARA, Selangor, Malaysia; McMaster University, Canada

## Abstract

**Background:**

Dengue virus-host cell interaction initiates when the virus binds to the attachment receptors followed by endocytic internalization of the virus particle. Successful entry into the cell is necessary for infection initiation. Currently, there is no protective vaccine or antiviral treatment for dengue infection. Targeting the viral entry pathway has become an attractive therapeutic strategy to block infection. This study aimed to investigate the effect of silencing the GRP78 and clathrin-mediated endocytosis on dengue virus entry and multiplication into HepG2 cells.

**Methodology/Principal Findings:**

HepG2 cells were transfected using specific siRNAs to silence the cellular surface receptor (GRP78) and clathrin-mediated endocytosis pathway. Gene expression analysis showed a marked down-regulation of the targeted genes (87.2%, 90.3%, and 87.8% for GRP78, CLTC, and DNM2 respectively) in transfected HepG2 cells when measured by RT-qPCR. Intracellular and extracellular viral RNA loads were quantified by RT-qPCR to investigate the effect of silencing the attachment receptor and clathrin-mediated endocytosis on dengue virus entry. Silenced cells showed a significant reduction of intracellular (92.4%) and extracellular viral RNA load (71.4%) compared to non-silenced cells. Flow cytometry analysis showed a marked reduction of infected cells (89.7%) in silenced HepG2 cells compared to non-silenced cells. Furthermore, the ability to generate infectious virions using the plaque assay was reduced 1.07 log in silenced HepG2 cells.

**Conclusions/Significance:**

Silencing the attachment receptor and clathrin-mediated endocytosis using siRNA could inhibit dengue virus entry and multiplication into HepG2 cells. This leads to reduction of infected cells as well as the viral load, which might function as a unique and promising therapeutic agent for attenuating dengue infection and prevent the development of dengue fever to the severe life-threatening DHF or DSS. Furthermore, a decrease of viremia in humans can result in the reduction of infected vectors and thus, halt of the transmission cycle.

## Introduction

Monocytes and macrophages have been considered as the primary targets of dengue virus (DENV) infection and are responsible for replication and dissemination of the virus after the onset of infection [1], [2]. Recent studies have also shown that the liver is an additional major target of DENV as supported by many evidences, including hepatomegaly, liver dysfunction [3], [4], [5], pathological findings [4], [6], [7], [8], [9], presence of viral antigens and DENV RNA in hepatocytes and Kupffer cells [10], [11], and virus recovery from liver biopsies [12]. Furthermore, different studies suggested that the severity and mortality of dengue infection were related to the involvement of hepatic abnormality and liver dysfunction in dengue hemorrhagic fever (DHF) and dengue shock syndrome (DSS) [3], [4], [6].

The infectious entry of DENV into the target cells is critical to establish the infection and is mediated by the viral E glycoprotein in both attachments and internalization into the host cells [13], [14], [15], [16], [17]. It comprises virion attachment to the cellular surface receptor, internalization into the cytoplasm by endocytosis, and finally release of nucleocapsid into the cytoplasm [18]. Currently multiple cell surface molecules were involved in DENV binding to the target cells. Previous studies have implicated glucose regulating protein 78 (GRP78) as a receptor on HepG2 cells (Hepatocytes) for DENV-2 entry [19], [20], [21]. GRP78, a stress-induced endoplasmic reticulum (ER) chaperone, is expressed at basal levels in normal adult organs such as the brain, lung, and liver. It is also reported on other cells such as proliferating endothelial and monocytic cells, but it is overexpressed on the membrane of malignant cells [22], [23], [24], [25], [26]. The critical role of GRP78 in the unfolded protein response as a part of the ER protein folding machinery has been well characterized [27]. It is involved in major biological functions of the regulation of protein folding and assembly, protein quality control, calcium binding and regulating ER stress signaling, intracellular protein trafficking [28], potent anti-apoptotic protein [29], [30], cell surface receptor-mediated endocytosis [31], and as a cell-surface protein that functions as a receptor in a range of cells [32]. Clathrin-mediated endocytosis has been identified as the main endocytic entry pathway for DENV [33], [34], [35]. Clathrin-mediated endocytosis pathway plays an essential role in the formation of coated vesicles, nutrient acquisition, clearance of apoptotic cells, antigen presentation, pathogen entry, receptor regulation, hypertension, and synaptic transmission.

RNA interference (RNAi) is a potent sequence-selective post-transcriptional gene control mechanism [36] and is mediated by small interfering RNAs (siRNA) [37], [38]. It has the advantage of significantly enhanced potency, specificity, and versatility compared to other traditional gene silencing methods [39], [40]. Since the first report on RNAi-mediated inhibition of respiratory syncytial virus (RSV) [41], several proof-of-concept studies have shown that pre-treatment or co-treatment with a virus-specific siRNAs can be used to inhibit the expression and/or replication of numerous viruses *in vitro* and *in vivo* [42] including HIV [43], HBV [44], [45], HCV [44], [46], WNV [47], CMV [48], and influenza virus [49]. Similarly, down regulation of cellular entry mechanisms using RNAi has a potential inhibitory effect on the DENV entry and multiplication into the target cells [50], [51], [52]. We observed previously that silencing CD14 associated molecule and clathrin-mediated endocytosis in human monocytes could inhibit entry and multiplication of DENV [50]. Thus, this study was proposed to use RNAi to attenuate dengue infection based on this observation as well as on the evidences that have showed the critical role of hepatocytes in dengue infection and progression to the severe life-threatening form of the disease [10], [53], [54], [55]. This was achieved by specifically silencing the GRP78 as an attachment receptor in HepG2 cells. In addition, two main components of the clathrin-mediated endocytosis pathway have also been carefully chosen; Clathrin heavy polypeptide (CLTC) which is required for clathrin-coated pits formation, and Dynamin 2 (Human-DNM2) which is essential for pinching off the endocytic vesicles from the plasma membrane.

## Materials and Methods

### Cells and virus

HepG2 was purchased from the American Type Culture Collection (cat #: HB-8065, ATCC, USA). Cells were maintained in DMEM supplemented with 10% FBS, 3.8 g/L sodium bicarbonate, 100 U/mL penicillin, and 100 µg/mL streptomycin at 37°C in 5% CO_2_. The experiments were performed using HepG2 cells with passage number between 10 and 35.

DENV-2 strain New Guinea C (NGC) was propagated in C6/36 cells (cat #: CRL-1660, ATCC, USA) and stored at −80°C until used as described previously [56]. Virus was titrated using the plaque assay on porcine kidney cells (PS clone D) as described previously [57]. PS clone D cells were sourced from Department of Medical Microbiology, Faculty of Medicine, University Malaya.

### siRNA design and synthesis

The nucleotide sequences of the GRP78 (NM_005347), CLTC (NM_004859), and DNM2 (NM_001005360) transcripts were retrieved from GenBank. siRNA sequences were designed by using the web-based tool IDT SciTools RNAi Design available from Integrated DNA Technologies (IDT), Inc. at www.idtdna.com. Three siRNAs were designed for each gene (Table 1). The specificity of the designed siRNA to the target gene is confirmed by BLAST searching online at (http://www.ncbi.nlm.nih.gov/BLAST) [58]. A pool consists of the three-siRNA oligonucleotides for each gene was custom chemically synthesized by 1^st^ BASE Pte Ltd, Singapore. One pre-validated siRNA targeting GAPDH gene as a positive siRNA control and one scramble siRNA as negative siRNA control were included in this study. The synthesized siRNAs were purified by HPLC, and a 2′-O-methyl modification at position 2 was introduced to deactivate the off-target activity of the siRNA without compromising the silencing effectiveness [59].

**Table 1:** Sequences of siRNA oligonucleotide template.

siRNA	Nucleotide sequence (sense strand)	Position^a^
**GRP 78 (1)**	5′r(GAAGGUUACCCAUGCAGUUGUUACT)3′	750–774
**GRP 78 (2)**	5′r(AGAUGAAGCUGUAGCGUAUGGUGCT)3′	1431–1455
**GRP 78 (3)**	5′r(CCACCAAGAUGCUGACAUUGAAGAC)3′	2082–2106
**CLTC (1)**	5′r(AGCCAGGACCCAGAUGUFC)3′	2607–2625
**CLTC (2)**	5′r(AUGUAUGAUGCUGCUAAGU)3′	4071–4089
**CLTC (3)**	5′r(CUCCACCAAUGACCUUAGA)3′	7964–7982
**DNM2 (1)**	5′r(GAAGGACAUCCGUGCAGCACUGGCA)3′	925–949
**DNM2 (2)**	5′r(GUACCAGUAAGCUCAGUUCCUACCC)3′	1515–1539
**DNM2 (3)**	5′r(CCCUUGACACCAUCCUGAAUGAGGG)3′	3075–3099
**GAPDH**	5′r(GAACAUCAUCCCUGCCUCUACUGGC)3′	714–738
**Scramble** b	5′r(ATGGACAGAATAAATGGACTT)3′	

Three siRNAs for each target gene were designed. The experiment includes also one Positive control siRNA for GAPDH gene and one scramble siRNA.

a The position refers to the siRNA sequence position on the target gene.

b Scrambled siRNA is a control used to markup any changes to the gene expression profile that may result from the siRNA delivery method.

### siRNA transfection

HepG2 cells were transfected using DharmaFECT-4 siRNA Transfection Reagent (Thermo Fisher Scientific, USA) by reverse transfection method in 24-well plate. For each well, suspension of 1×10^5^ cells were mixed with a mixture of siRNA-transfection reagent complex to deliver final concentrations of 50 nM of each GRP78, CLTC and DNM2 siRNA. The positive control was transfected with pre-validated siRNA targeting GAPDH gene. The negative control received the DharmaFECT-4 Transfection Reagent plus the scramble siRNA. For monitoring the gene silencing effect, cells were harvested after 24 hours; while for evaluating the viral entry after infection with DENV-2, cells were harvested after 72 hours.

### Cytotoxicity

Cytotoxicity was tested using CytoTox-ONE™ Homogeneous Membrane Integrity Assay (Promega, USA) that measure LDH released from cells with compromised membrane in accordance to the manufacturer's protocol.

### Gene knockdown verification

Gene expression analysis of the target genes was performed by RT-qPCR usingCFX96™ Real-Time PCR Detection System (Bio-Rad, USA). Primer sets for GRP78, CLTC, DNM2, RPL27, and GAPDH were designed by using Primer Express software V3.0. Primer set for RPS29 was obtained from published sequences [60] (Table 2). A pair of primer was considered valid when the efficacy of amplification is between 90–110% with a minimum R^2^ of 0.980. Reference genes for this study were identified by using geNorm v3.5 software [61]. The RT-qPCR protocol and reaction conditions were carried out as described previously [50]. RT-qPCR experiments were performed in three technical replicates, and no template control and no-reverse transcription control were also included. Baseline and quantification cycle (Cq) values and gene expression analysis were automatically analyzed using the Bio-Rad CFX Manager Software 1.6.

**Table 2:** Primer sequences.

Accession number	Gene symbol	Forward sequence (5′→3′)	Reverse sequence (5′→3′)	(bp)
**(NM_005347)**	GRP78	ACTATGAAGCCCGTCCAGAA	GACAGCAGCACCATACGCTA	189
**(NM_004859)**	CLTC	CCACAATACTCCACCAATGACCTTA	CCCATTTTAAGTGGCTCCACCTCTC	97
**(NM_001005360)**	DNM2	GGCATTCGAGGCCATTGT	CATTTCAGACAGGGCTCTTTCA	60
**(NM_002046)**	GAPDH	CATCACCATCTTCCAGGAGCG	TGATGGCATGGACTGTGGTC	326
**(NM_000988)**	RPL27	CATGGGCAAGAAGAAGATCG	ACGACAGTTTTGTCCAAGGG	120
**(NM_001030001)**	RPS29	GCACTGCTGAGAGCAAGATG	ATAGGCAGTGCCAAGGAAGA	213
**(NC001477)**	DENV_NS5	GGAAGGAGAAGGACTGCACA	ATTCTTGTGTCCCATCCTGCT	104

Primer sequences of the target genes, positive control gene, optimal reference genes and DENV NS5.

### HepG2 cells infection

Infection of silenced and non-silenced HepG2 cells was performed at a density of 1×10^5^ cells in a 24-well plate as described previously [50]. After 72 hours, cellular supernatant were collected and stored in aliquot at −80°C until use for infectious virion titration by plaque assay and viral RNA copies quantification by RT-qPCR while cells were harvested for counting the infected cells by flow cytometry and viral RNA quantification by RT-qPCR.

### Viral RNA quantification

One-step RT-qPCR was carried out in CFX96™ Real-Time PCR Detection System using the iScript™ One-Step RT-PCR Kit with SYBR® Green as described previously [50]. Three technical replicates were carried out for each sample and no template control was included as a negative control.

### Flow cytometry analysis

Silenced and non-silenced DENV-2 infected and non-infected HepG2 cells (control) were harvested for flow cytometric quantification of DENV infected cells as described previously [50], [62]. Flow cytometry analysis was performed using indirect staining in which monoclonal antibody (anti-DENV-2 for detection of positive samples and anti-DENV-3 as a negative control) was used as a primary antibody. The cells were then incubated with a FITC-labeled goat anti-mouse IgG as a secondary antibody. During flow cytometry, 100,000 events were acquired on a FACScaliber using Cell Quest software (Becton Dickinson Immunocytometry System, CA). The percentage of positive cells and the average fluorescence intensities were determined from FITC fluorescence histogram using a region that was defined based on analysis of the infected control.

### Statistical analysis

Cytotoxicity assay, gene expression analysis, HepG2 transfection and infection, flow cytometry detection of infected cells, and intracellular and extracellular quantification of viral RAN by RT-qPCR were done at least in three biological experiments. All statistical analyses were performed using GraphPad Prism version 5.01 (GraphPad Software, USA). P values<0.05 were considered significant. Results are expressed as mean ± SD from a representative experiment performed in triplicate.

## Results

### Cytotoxicity and optimization of siRNA transfection

**Figure 1:**
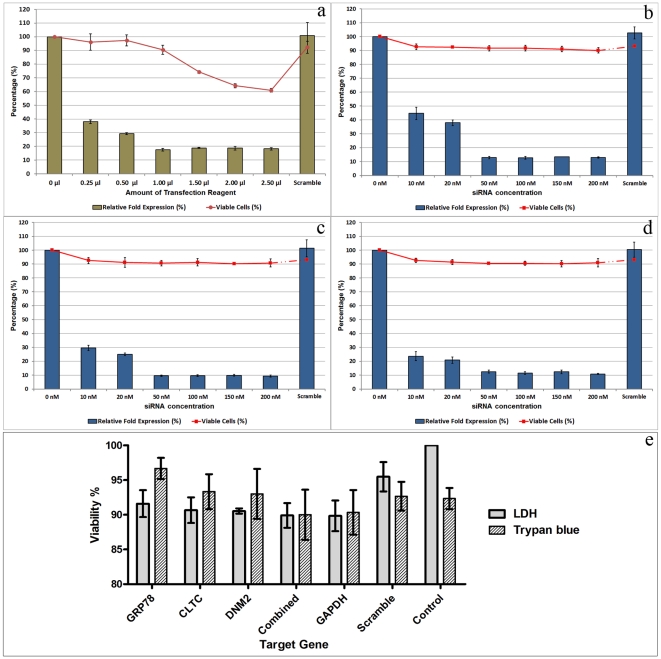
Cytotoxicity and optimization of siRNA transfection. HepG2 cells transfection experiment was optimized by treating the HepG2 cells with increasing amount of transfection reagent and concentrations of siRNA. (a) Result shows that the efficient silencing with minimal cytotoxicity was achieved using 1.0 µl of transfection reagent. (b) HepG2 cells transfected with increased concentration of GRP78 siRNAs and the result shows no evidence of toxicity regardless of the siRNA concentration as compared with scramble siRNA transfected HepG2 cells (One-way ANOVA with Dunnett's post-test, P>0.05). The optimal siRNA concentration is 50 nM. Similar results were observed in (c) CLTC siRNAs and (d) DNM2 siRNAs. (e) HepG2 cells were transfected and exposed to optimal siRNAs concentration (50 nM) that target GRP78, CLTC, and DNM2 in separated and combined transfection. Cytotoxicity was tested by measuring LDH level and results were confirmed by counting of viable cells using Trypan Blue exclusion assay. No evidence of cytotoxicity was observed for all pools of siRNA as well as in combined transfection (91.6%±2.0, 90.7%±1.9, 90.5%±0.4, and 89.9%±1.8 for GRP78, CLTC, DNM2, and combined transfection respectively). Trypan Blue exclusion assay showed also more than 90% viable cells. No significant difference was observed between separated and combined transfection by One-way ANOVA analysis (P>0.05). Results are expressed as mean ± SD from a representative experiment performed in triplicate.

The transfection experiment was optimized to produce maximum silencing with minimal or no cytotoxicity. Cytotoxicity was determined by measuring the levels of released LDH from cells with compromised membrane. Transfection experiment was optimized by treating the HepG2 cells with increasing concentrations of siRNA and transfection reagent. Results show that efficient silencing with minimal cytotoxicity was achieved using 1 µl of transfection reagent. No evidence of toxicity was shown regardless of the concentration of siRNA compared with scramble siRNA transfected HepG2 cells (One-way ANOVA with Dunnett's post-test, P>0.05). The optimal siRNA concentration is 50 nM for GRP78, CLTC, and DNM2 (Figure 1).

After optimization, transfection experiments were carried out at a cell density of 1.0×10^5^ cells/well in a 24-well plate using 1.0 µL DharmaFECT-4 siRNA transfection reagent and siRNA concentration of 50 nM for GRP78, CLTC, and DNM2. This transfection condition showed a minimal toxicity effect on the transfected cells. The percentages of the viable cells were 91.6%±2.0, 90.7%±1.9, 90.5%±0.4, and 89.9%±1.8 for GRP78, CLTC, DNM2, and combined transfection respectively compared with non-transfected HepG2 cells (One-way ANOVA with Dunnett's post-test, P>0.05). This observation was further confirmed by counting the number of viable cells using Trypan Blue exclusion assay that showed more than 90% of the cells were viable as shown in Figure 1.

### Gene knockdown verification

**Figure 2:**
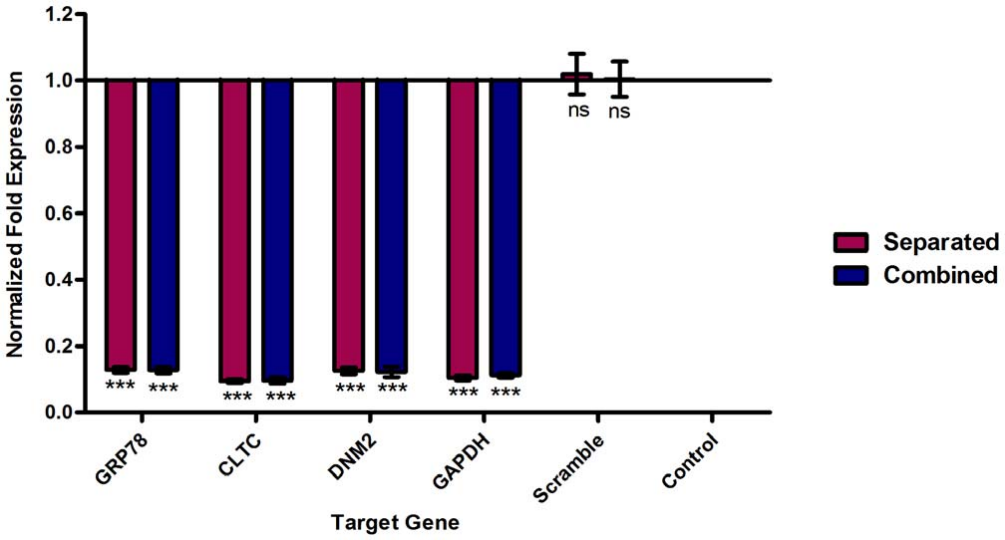
Efficiency of silencing target genes. Each of target genes was targeted with a specific pool of siRNA. An efficient gene silencing was achieved in both separated and combined transfection when compared with non-transfected control and normalized to reference genes (One-way ANOVA with Dunnett's post-test, P<0.0001, Results are expressed as mean ± SD from a representative experiment performed in triplicate). There are no significant differences between separated transfection (87.1%±0.9, 90.5%±0.5, and 87.4%±1.0) and combined transfection (87.2%±1.0, 90.3%±0.9, and 87.8%±1.6) on gene expression of GRP78, CLTC, and DNM2 respectively (Two-way ANOVA with Bonferroni post-test, P>0.05, Results are expressed as mean ± SD from a representative experiment performed in triplicate). Scrambled siRNA control had no inhibitory effect on any gene expression, and a similar expression to the non-transfected control was observed (One-way ANOVA with Dunnett's post-test, P>0.05).

The effect of siRNA transfection on related gene expression was investigated by RT-qPCR using mRNA extracted from the transfected HepG2 cells. Gene expression results were shown as normalized-fold expression compared to non-transfected control. Firstly, the silencing efficiency of the pooled siRNAs, which comprised of the three different siRNAs targeting the same gene, was screened. As shown in Figure 2, the knockdown levels were 87.1%±0.9, 90.5%±0.5, and 87.4%±1.0 for pooled siRNA of GRP78, CLTC, and DNM2 respectively (One-way ANOVA with Dunnett's post-test, P<0.0001). HepG2 cells were then co-transfected with a combination of the three different siRNA pools to target the three genes simultaneously. Result showed no significant differences in the knockdown level for all the genes (87.2%±1.0, 90.3%±0.9, and 87.8%±1.6 for GRP78, CLTC, and DNM2 respectively) when compared to separated transfection (Two-way ANOVA with Bonferroni post-test, P>0.05). Furthermore, no inhibitory effect on any gene expression was observed in the scramble siRNA transfected HepG2 cells as shown in Figure 2 (One-way ANOVA with Dunnett's post-test, P>0.05).

### Quantification of infected HepG2 cells

**Figure 3:**
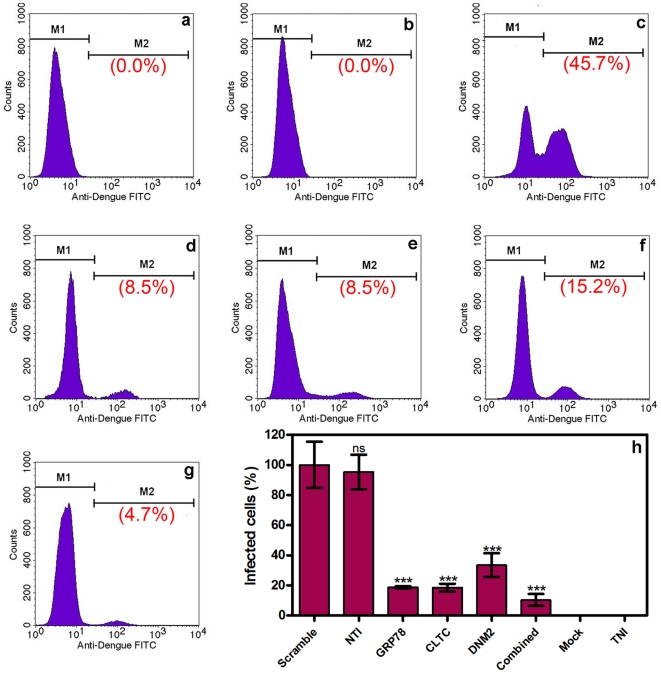
Quantification of infected cells by flow cytometry. Silenced and non-silenced HepG2 cells were infected by DENV-2 at MOI of 2. Result showed a marked reduction in percentage of infected cells by flow cytometry. This figure shows the percentage of DENV infected cells at different conditions. (a) Transfected non-infected HepG2 cells (0.0%) represent the negative control. (b) Transfected mock-infected HepG2 cells as a staining control (0.0%). (c) Scramble siRNA transfected infected HepG2 cells (45.7%) as a positive control. (d) GRP78 silenced infected HepG2 cells (8.5%). (e) CLTC silenced infected HepG2 cells (8.5%). (f) DNM2 silenced infected HepG2 cells (15.2%). (g) GRP78, CLTC, and DNM2 combined silenced infected HepG2 cells (4.7%). (h) Summarized the results of the flow cytometry experiments. Data is expressed as a percentage of infected cells compared with scramble siRNA transfected infected HepG2 cells which was defined as 100%. The percentages of the infected cells are 18.7%±0.7, 18.6%±2.6, 33.4%±7.9, and 10.3%±3.9 for GRP78, CLTC, DNM2, and combined silenced HepG2 cells respectively (One-way ANOVA with Dunnett's post-test, P<0.0001, Results are expressed as mean ± SD from a representative experiment performed in triplicate). Also, statistical analysis shows no significant difference between scramble siRNA transfected and non-transfected infected HepG2 cells (One-way ANOVA with Dunnett's post-test, P>0.05).

Dengue infected HepG2 cells were quantified to determine whether silencing of GRP78 and/or clathrin endocytosis pathway could inhibit DENV entry into HepG2 cells and, therefore, reduce its multiplication. Both silenced and non-silenced HepG2 cells were infected with DENV-2 at a MOI of 2. Result showed a marked reduction in the percentage of infected cell in GRP78, CLTC, and DNM2 silenced HepG2 by using flow cytometry. The percentage of infected cells was reduced from 45.7%±7.0 in scramble siRNA transfected HepG2 cells to 8.5%±0.3 (81.3%), 8.5%±1.2 (81.4%), and 15.2%±3.6 (66.6%) in GRP78, CLTC, and DNM2 silenced HepG2 cells respectively. Interestingly, combined silencing (GRP78, CLTC, and DNM2) of HepG2 cells showed a higher inhibitory effect on DENV entry and replication (4.7%±1.8) which represented 89.7% reduction in infected cells compared to scramble siRNA transfected HepG2 cells as shown in Figure 3 (One-way ANOVA with Dunnett's post-test, P<0.0001). Furthermore, statistical analysis of the results shows no significance difference between the scramble siRNA transfected and non-transfected infected HepG2 cells (One-way ANOVA with Dunnett's post-test, P>0.05).

### Quantification of intracellular viral RNA load

**Figure 4:**
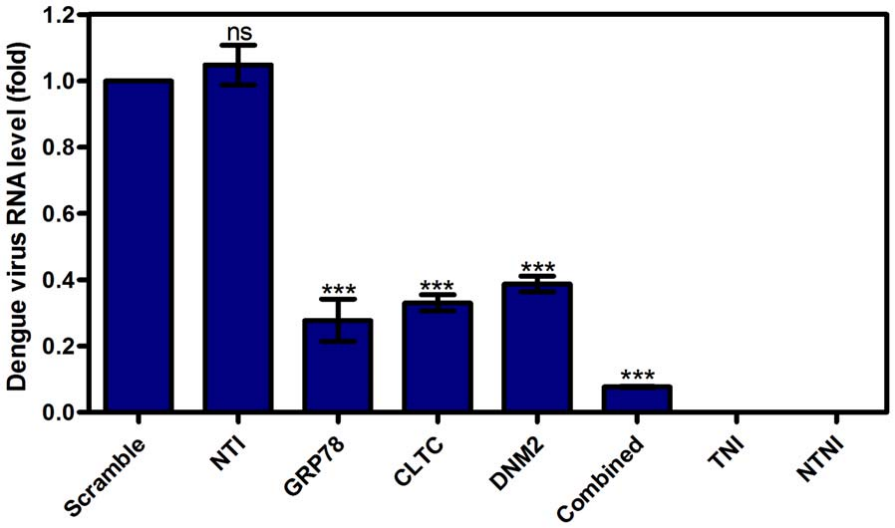
Quantification of intracellular dengue virus RNA load. Viral RNA levels were quantified by RT-qPCR and normalized to reference gene (RPL27). Data is expressed as relative-fold expression to scramble siRNA transfected HepG2 cells control, which defined as 1.0 fold. Intracellular viral RNA load reduced (0.72 fold ±0.11), (0.67 fold ±0.04), (0.61 fold ±0.04), and (0.92 fold ±0.004) in GRP78, CLTC, DNM2, and combined silenced HepG2 cells respectively (One-way ANOVA with Dunnett's post-test, P<0.0001, Results are expressed as mean ± SD from a representative experiment performed in triplicate). No significant difference between scramble siRNA transfected and non-transfected infected HepG2 cells (One-way ANOVA with Dunnett's post-test, P>0.05). (TNI, Transfected Non-Infected; NTI, Non-Transfected Infected; NTNI, Non-Transfected Non-Infected).

The reduction of the percentage of infected cell in silenced HepG2 cells was further confirmed by quantification of intracellular viral RNA load using RT-qPCR analysis. The viral RNA load in silenced HepG2 cells was compared to the scramble siRNA transfected HepG2 cells and was normalized to the reference gene (RPL27). Data is expressed as relative-fold expression to scramble siRNA transfected HepG2 cells, which was defined as 1.0 fold (viral RNA copy number is 1.21×10^4^/µL). Result showed a significant reduction of DENV RNA level in silenced HepG2 cells: 0.72 fold ±0.11, 0.67 fold ±0.04, 0.61 fold ±0.04, and 0.92 fold ±0.004 in GRP78, CLTC, DNM2, and combined silenced HepG2 cells respectively as shown in Figure 4 (One-way ANOVA with Dunnett's post-test, P<0.0001). Again, there is no significant difference between the scramble siRNA transfected and non-transfected infected HepG2 cells (One-way ANOVA with Dunnett's post-test, P>0.05).

### Quantification of viral RNA load and plaque forming units in culture supernatant

**Figure 5:**
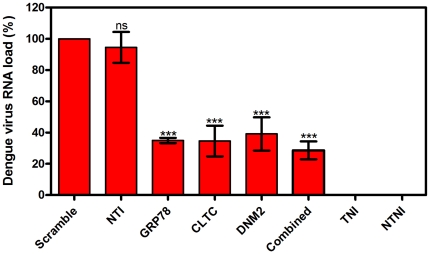
Quantification of extracellular dengue virus RNA load. RT-qPCR was used to quantify the DENV RNA in the culture supernatant of silenced and non-silenced HepG2 cells. Marked reduction in viral RNA was achieved (65.1%±1.7), (65.4%±9.8), (60.9%±10.6), and (71.4%±5.7) for GRP78, CLTC, DNM2, and combined silenced HepG2 cells respectively. This result is statistically significant (One-way ANOVA with Dunnett's post-test, P<0.0001, Results are expressed as mean ± SD from a representative experiment performed in triplicate). There is no significant difference between scramble siRNA transfected and non-transfected infected HepG2 cells (One-way ANOVA with Dunnett's post-test, P>0.05). (TNI, Transfected Non-Infected; NTI, Non-Transfected Infected; NTNI, Non-Transfected Non-Infected).

**Figure 6:**
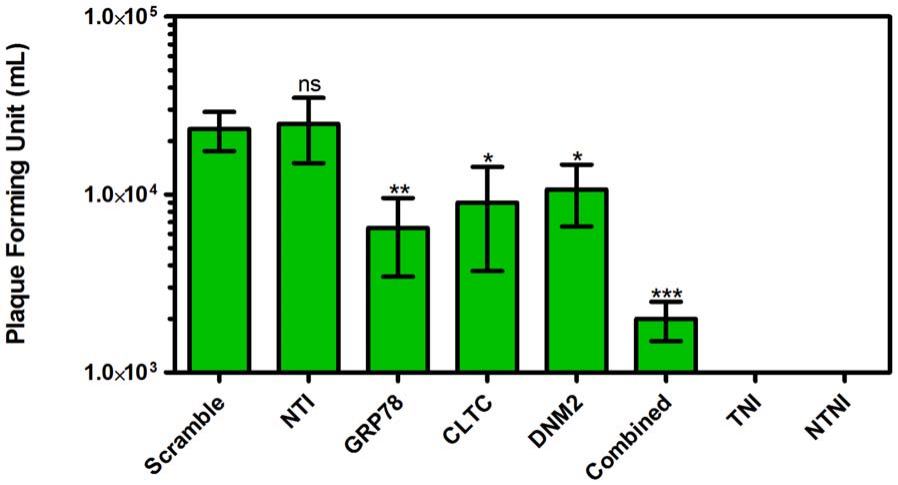
Quantification of plaque forming infectious virions in culture supernatant. The ability to produce infectious virions was investigated by plaque assay. Figure shows marked reduction of the plaque forming units (0.56 log), (0.41 log), (0.34 log), and (1.07 log) for GRP78, CLTC, DNM2, and combined silenced HepG2 cells respectively compared to scramble siRNA transfected cells. All plaques were counted at day 7 of incubation. Plaques are expressed as plaque forming unit per mL (pfu/mL). This result is statistically significant (One-way ANOVA with Dunnett's post-test, P<0.0001, Results are expressed as mean ± SD from a representative experiment performed in triplicate) and there is no significant difference between scramble siRNA transfected and non-transfected infected HepG2 cells (One-way ANOVA with Dunnett's post-test, P>0.05). (TNI, Transfected Non-Infected; NTI, Non-Transfected Infected; NTNI, Non-Transfected Non-Infected).

DENV excreted from the silenced HepG2 cells into the culture supernatant was quantified by RT-qPCR and compared to scramble siRNA transfected HepG2 cells. Result showed a similar observation when compared to the intracellular quantification of viral RNA. Figure 5 described the DENV RNA load in the culture supernatant of silenced HepG2 cells as compared to scramble siRNA transfected HepG2 cells, which was defined as 100% (viral RNA copy number is 6.50×10^4^/µL). The reduction in viral RNA was 65.1%±1.7, 65.4%±9.8, 60.9%±10.6, and 71.4%±5.7 for GRP78, CLTC, DNM2, and combined silenced HepG2 cells respectively. This result is statistically significant (One-way ANOVA with Dunnett's post-test, P<0.0001). To confirm the previous results, DENV virus titer was determined by plaque assay from the harvested culture medium 72 hours post infecting the transfected HepG2 cells. Plaques were counted at day 7 of incubation. Plaques are expressed as plaque forming unit per mL (pfu/mL). Result showed a dramatically reduction in the ability to generate infectious virions in silenced HepG2 cells. The reduction in the plaque forming units was 0.56, 0.41, and 0.34 log in separated silencing of GRP78, CLTC, DNM2 respectively. Furthermore, the titer of the plaque forming units was reduced up to 1.07 log in the combined silencing of GRP78, CLTC, and DNM2 in HepG2 cells compared to scramble siRNA transfected HepG2 cells as shown in Figure 6 (One-way ANOVA with Dunnett's post-test, P<0.0005).

## Discussion

The present study investigates the effects of siRNA on suppression the dengue infection in HepG2 cells by silencing the GRP78, CLTC, and DNM2 genes separately or in combination. The silenced HepG2 cells with specific siRNA duplexes (GRP78, CLTC, and DNM2) revealed a reduction of the percentage of infected cells as shown by flow cytometry analysis, reduction of the intracellular and extracellular viral RNA load when monitored by RT-qPCR, and reduction of generation of infectious virions as observed by plaque assay. The findings have shown that the suppression was specific and efficient. This results are consistent with our previous findings of inhibition of dengue infection in monocytes by silencing the CD-14 associated molecule and clathrin-mediated endocytosis [50]. Our previous study has showed a significant reduction of infected cells (85.2%), intracellular viral RNA load (73.0%), and extracellular viral RNA load (63.0%) in silenced monocytes as compared to non-silenced monocytes. As seen in the current study, silenced HepG2 cells showed a more efficient reduction of infected cells (89.7%), intracellular viral RNA load (92.4%), and extracellular viral RNA load (70.7%) compared to monocytes. This variation could be attributed to the more effectiveness of the gene silencing in HepG2 cells than in monocytes as well as the monocytes are primary cells, whereas the HepG2 cells are a cell line.

In dengue infection, evidences have shown that the liver is a major target organ for DENV infection in humans as many pathological findings and liver dysfunction have been detected in the livers of DHF/DSS patients and is a characteristic of severe dengue infection [10], [53], [54], [55]. It has been demonstrated that there is a direct correlation between the development of the severe and life-threatening form of the disease and high viral load [63]. Hepatocytes were considered as a target in this study to reduce the dengue viral load during the course of infection based on evidences of its crucial role in dengue infection, and the previous findings of the inhibition of dengue infection in monocytes. Prevention the multiplication of DENV in the main target cells could result in reduction of the total viral load during the course of infection. This would potentially prevent the progression of dengue fever to the severe life-threatening form of dengue infection. Furthermore, a decreased of viremia in humans can result in the drop in number of infected vectors and, therefore, break of the transmission chain.

There are various strategies to develop antiviral agents against DENV. Recently, the virus entry step has become an attractive therapeutic strategy [64]. DENV-host cell interaction is initiated with the virus binding on attachment receptors on the cell surface followed by stimulation of signals that result in the endocytic internalization of the virus particles. This is critical for successful entry into the host cell and the establishment of infection. This study was designed to target the GRP78 that has been identified as receptor on HepG2 cells for DENV-2 entry [19], [20], [21], and clathrin-mediated endocytosis that known as the main pathway for virus internalization [65]. This is a possible way to provide a new therapeutic strategy by targeting the host factors known to be involved in viral infection. Thus, it is expected to control viral infection by making the cellular receptors for viruses on human cells less accessible. Recent studies showed that design and synthesis of agents that prevent DENV binding and entry to the cellular receptor sites could prove to be novel antiviral agents of preventing the disease. RNAi pathway shows a role in modulating DENV replication in previous studies [50], [51], [52].

In the present study, the designed siRNA showed an efficient knockdown of the target genes when evaluated at the mRNA level 24 hours after transfection. To evaluate the viral entry into HepG2 cells, the silenced cells were infected with a live DNEV 72 hours post transfection to confirm that the target proteins level was reduced as the half-life time of these target proteins is less than 72 hours as shown by previous studies. These proteins possess a half-life of 24–48 hours for GRP78 [66], [67], [68], [69], [70], [71], 18–36 hours for CLTC [72], [73], [74], and 24–34 hours for DNM2 [75].

Silencing the GRP78 and clathrin-mediated endocytosis resulted in significant inhibition of dengue infection up to 81.3% and 81.4% for GRP78 and the clathrin-mediated endocytosis respectively. In addition, a more potent inhibition (up to 89.7%) is observed when a combined silencing of both GRP78 and clathrin-mediated endocytosis was done simultaneously. This reduction in viral yield was not due to cell death as could be identified by cytotoxicity and viability tests (>90% live cells) in both silenced and non-silenced HepG2 cells. GRP78 could be up regulated in dengue infected cells as a direct response to productive infection in dengue infected cells and as a secondary consequence, in both dengue infected and bystander cells, of the release of cytokines and factors from dengue infected cells that can induce GRP78 expression. Previous studies have suggested two roles for GRP78 in dengue infection. First, GRP78 serves as part of a receptor complex for DENV entry into hepatocytes [19], [20], [21]. Additionally, heat shock treatment of cells in the tissue culture system prior to DENV challenge, which would be expected to up regulate both GRP78 and HSP70, thus enhancing DENV entry and replication [76]. Second, GRP78 is known to function as a major ER chaperone and master regulator of unfolded protein responses [77]. Dengue infection and viral protein production results in ER stress [78]. An overwhelming load of misfolded proteins up regulate the GRP78 [79]. Increasing the requirement of GRP78 triggers a signaling of unfolded protein response (UPR) pathway [80]. Thus, the UPR pathway restores the normal function of the cell by enhancing transcription of ER chaperones, decreasing protein translation to mitigate the ER overload, increasing protein degradation, or activates the apoptotic cell death [78], [81], [82]. In this study, silencing GRP78 showed inhibition of dengue infection in HepG2 cells. Based on above explanation, we propose that GRP78 inhibits dengue infection either by interaction with cell-surface attachment and internalization and then multiplication. The other possible way is that GRP78 may function in its traditional role in binding and chaperoning many unfolded proteins, include DENV proteins [83]. Our result is consistent with a previous observation showing that cleaving the GRP78 by SubAB toxin can dramatically reduce the releasing of infectious DENV and intracellular virion particles [84]. Another study also shows the siRNA knockdown of GRP78 in HepG2 cells decreased infectious-virus production [85]. Furthermore, elimination of GRP78 leads to reduction of cytosolic ubiquitination and inhibits ubiquitination-proteosome pathway which is known to regulate the endocytosis of cell-surface receptors [86], [87]. Therefore, inhibiting the cellular endocytosis pathway possibly leads to inhibit the internalization of DENV [14], [51] and other flaviviruses [88].

Incomplete inhibition of dengue infection can be interpreted as the result of using an alternative secondary receptor or endocytosis by the DENV to establish the infection or incomplete inhibition by the RNAi machinery. For RNAi based therapy designing, it is necessary to consider that this technology knockdown gene expression, but in general does not eliminate it. Therefore, in some conditions, incomplete down regulation of a pathogenic gene seems to be adequate to produce a clinically appropriate improvement [40].

The approach of gene silencing is a widely accepted technique and, recently, RNAi technique had shown a potential to achieve the gene therapy goal [89]. The effectiveness of siRNA as a therapy against dengue infection will depend on the efficiency of siRNA delivery to the target mammalian cells [90]. However, for application of RNAi as a dengue therapeutic, an effective and cell-specific *in vivo* delivery system is required. Recently, several studies have described a success in siRNAs delivery, *in vivo*, by coupling to antibodies or peptides that recognize cell surface receptors [91]. This can provide a further supporting and future potential for the practical utility of this approach. The aim of any therapeutic is to maximize the ratio of desired effects to undesired effects. In some cases, such as chemotherapy, interferon treatment, and highly active anti-retroviral treatment, the ratio is not ideal and a significant degree of toxicity is associated with treatment. While RNAi has the capacity to provide better gene targeting specificity, the exposure of cells to any exogenous molecule (siRNA or transfecting reagent) has the potential to disturb normal cellular functions and needs to be carefully controlled. In this study, the transfection experiment was optimized to produce maximum silencing effect at low-cell toxicity as revealed by measurement the released LDH from cells with compromised membrane and Trypan Blue exclusion assays in cell viability test.

One of the potential weaknesses of the RNAi based therapeutic is the problem of resistance and RNAi escape mutations [40]. This problem will probably require the use of RNAi in combination therapy approaches, including multiple RNAi target sequences and/or other synergistic antiviral drugs as well as by targeting the host factors known to be involved in viral infection, such as entry receptor or binding molecules on the susceptible cells. Here, we used three different siRNA pools targeting GRP78, CLTC, and DNM2. Each pool achieved its own silencing level and similar knockdown efficiency of these genes in HepG2 cells was achieved by co-transfection of the three pools simultaneously. The desire to pool three siRNAs was raised primarily from the finding that the silencing efficiency of a single siRNAs was lesser than combined pool of triple siRNAs. These pools have shown a greater potency in the reduction of the target gene expression and elimination of off-targets effect.

### Conclusions

We have successfully inhibited the DENV entry and multiplication into HepG2 cells by silencing the GRP78 and clathrin mediated endocytosis. Reduction of the viral load would potentially prevent dengue fever and the severe life-threatening form of dengue infection. Decreasing viremia in humans can result in a decline in the infected vectors numbers, and thus interruption of the transmission chain. This might not only save lives but also curb potential epidemics. This tool might serve as a novel promising therapeutic agent for the attenuation of dengue infection and reduction the progression to severe form of the disease DHF.

## References

[1] Halstead SB (1989). Antibody, macrophages, dengue virus infection, shock, and hemorrhage: a pathogenetic cascade.. Rev Infect Dis.

[2] Chen YC, Wang SY, King CC (1999). Bacterial lipopolysaccharide inhibits dengue virus infection of primary human monocytes/macrophages by blockade of virus entry via a CD14-dependent mechanism.. J Virol.

[3] Nguyen TL, Nguyen TH, Tieu NT (1997). The impact of dengue haemorrhagic fever on liver function.. Res Virol.

[4] Wahid SF, Sanusi S, Zawawi MM, Ali RA (2000). A comparison of the pattern of liver involvement in dengue hemorrhagic fever with classic dengue fever.. Southeast Asian J Trop Med Public Health.

[5] Mohan B, Patwari AK, Anand VK (2000). Hepatic dysfunction in childhood dengue infection.. J Trop Pediatr.

[6] Bhamarapravati N, Tuchinda P, Boonyapaknavik V (1967). Pathology of Thailand haemorrhagic fever: a study of 100 autopsy cases.. Ann Trop Med Parasitol.

[7] Burke T (1968). Dengue haemorrhagic fever: a pathological study.. Trans R Soc Trop Med Hyg.

[8] Bhamarapravati N (1989). Hemostatic defects in dengue hemorrhagic fever.. Rev Infect Dis.

[9] Kurane I, Rothman AL, Livingston PG, Green S, Gagnon SJ (1994). Immunopathologic mechanisms of dengue hemorrhagic fever and dengue shock syndrome.. Arch Virol.

[10] Huerre MR, Lan NT, Marianneau P, Hue NB, Khun H (2001). Liver histopathology and biological correlates in five cases of fatal dengue fever in Vietnamese children.. Virchows Arch.

[11] Rosen L, Drouet MT, Deubel V (1999). Detection of dengue virus RNA by reverse transcription-polymerase chain reaction in the liver and lymphoid organs but not in the brain in fatal human infection.. Am J Trop Med Hyg.

[12] Rosen L, Khin MMUT (1989). Recovery of virus from the liver of children with fatal dengue: reflections on the pathogenesis of the disease and its possible analogy with that of yellow fever.. Res Virol.

[13] Chen Y, Maguire T, Marks RM (1996). Demonstration of binding of dengue virus envelope protein to target cells.. J Virol.

[14] Clyde K, Kyle JL, Harris E (2006). Recent advances in deciphering viral and host determinants of dengue virus replication and pathogenesis.. J Virol.

[15] Kuhn RJ, Zhang W, Rossmann MG, Pletnev SV, Corver J (2002). Structure of dengue virus: implications for flavivirus organization, maturation, and fusion.. Cell.

[16] Chen Y, Maguire T, Hileman RE, Fromm JR, Esko JD (1997). Dengue virus infectivity depends on envelope protein binding to target cell heparan sulfate.. Nat Med.

[17] Crill WD, Roehrig JT (2001). Monoclonal antibodies that bind to domain III of dengue virus E glycoprotein are the most efficient blockers of virus adsorption to Vero cells.. J Virol.

[18] Hung SL, Lee PL, Chen HW, Chen LK, Kao CL (1999). Analysis of the steps involved in Dengue virus entry into host cells.. Virology.

[19] Jindadamrongwech S, Thepparit C, Smith DR (2004). Identification of GRP 78 (BiP) as a liver cell expressed receptor element for dengue virus serotype 2.. Arch Virol.

[20] Upanan S, Kuadkitkan A, Smith DR (2008). Identification of dengue virus binding proteins using affinity chromatography.. J Virol Methods.

[21] Cabrera-Hernandez A, Thepparit C, Suksanpaisan L, Smith DR (2007). Dengue virus entry into liver (HepG2) cells is independent of hsp90 and hsp70.. Journal of Medical Virology.

[22] Lee AS (2001). The glucose-regulated proteins: stress induction and clinical applications.. Trends in Biochemical Sciences.

[23] Hendershot LM (2004). The ER function BiP is a master regulator of ER function.. The Mount Sinai journal of medicine, New York.

[24] Kiang JG, Tsokos GC (1998). Heat shock protein 70 kDa: Molecular biology, biochemistry, and physiology.. Pharmacology & Therapeutics.

[25] Davidson DJ, Haskell C, Majest S, Kherzai A, Egan DA (2005). Kringle 5 of human plasminogen induces apoptosis of endothelial and tumor cells through surface-expressed glucose-regulated protein 78.. Cancer Res.

[26] Bhattacharjee G, Ahamed J, Pedersen B, El-Sheikh A, Mackman N (2005). Regulation of tissue factor–mediated initiation of the coagulation cascade by cell surface grp78.. Arterioscler Thromb Vasc Biol.

[27] Schroder M, Kaufman RJ (2005). The mammalian unfolded protein response.. Annu Rev Biochem.

[28] Awe K, Lambert C, Prange R (2008). Mammalian BiP controls posttranslational ER translocation of the hepatitis B virus large envelope protein.. FEBS Lett.

[29] Li J, Lee B, Lee AS (2006). Endoplasmic reticulum stress-induced apoptosis: multiple pathways and activation of p53-up-regulated modulator of apoptosis (PUMA) and NOXA by p53.. J Biol Chem.

[30] Reddy RK, Mao C, Baumeister P, Austin RC, Kaufman RJ (2003). Endoplasmic reticulum chaperone protein GRP78 protects cells from apoptosis induced by topoisomerase inhibitors: role of ATP binding site in suppression of caspase-7 activation.. J Biol Chem.

[31] Liu Y, Steiniger SC, Kim Y, Kaufmann GF, Felding-Habermann B (2007). Mechanistic studies of a peptidic GRP78 ligand for cancer cell-specific drug delivery.. Mol Pharm.

[32] Delpino A, Castelli M (2002). The 78 kDa glucose-regulated protein (GRP78/BIP) is expressed on the cell membrane, is released into cell culture medium and is also present in human peripheral circulation.. Biosci Rep.

[33] Peng T, Wang JL, Chen W, Zhang JL, Gao N (2009). Entry of dengue virus serotype 2 into ECV304 cells depends on clathrin-dependent endocytosis, but not on caveolae-dependent endocytosis.. Can J Microbiol.

[34] van der Schaar HM, Rust MJ, Chen C, van der Ende-Metselaar H, Wilschut J (2008). Dissecting the cell entry pathway of dengue virus by single-particle tracking in living cells.. PLoS Pathog.

[35] Acosta EG, Castilla V, Damonte EB (2011). Infectious dengue-1 virus entry into mosquito C6/36 cells.. Virus Res.

[36] Fire A, Xu S, Montgomery MK, Kostas SA, Driver SE (1998). Potent and specific genetic interference by double-stranded RNA in Caenorhabditis elegans.. Nature.

[37] Tuschl T (2001). RNA interference and small interfering RNAs.. Chembiochem.

[38] Elbashir SM, Harborth J, Lendeckel W, Yalcin A, Weber K (2001). Duplexes of 21-nucleotide RNAs mediate RNA interference in cultured mammalian cells.. Nature.

[39] Le Calvez H, Yu M, Fang F (2004). Biochemical prevention and treatment of viral infections - a new paradigm in medicine for infectious diseases.. Virol J.

[40] Uprichard SL (2005). The therapeutic potential of RNA interference.. FEBS Lett.

[41] Bitko V, Barik S (2001). Phenotypic silencing of cytoplasmic genes using sequence-specific double-stranded short interfering RNA and its application in the reverse genetics of wild type negative-strand RNA viruses.. BMC Microbiol.

[42] Haasnoot PC, Cupac D, Berkhout B (2003). Inhibition of virus replication by RNA interference.. J Biomed Sci.

[43] Berkhout B (2004). RNA interference as an antiviral approach: Targeting HIV-1.. Current opinion in molecular therapeutics.

[44] Radhakrishnan SK, Layden TJ, Gartel AL (2004). RNA interference as a new strategy against viral hepatitis.. Virology.

[45] Arbuthnot P, Carmona S, Ely A (2005). Exploiting the RNA interference pathway to counter hepatitis B virus replication.. Liver Int.

[46] Randall G, Rice CM (2004). Interfering with hepatitis C virus RNA replication.. Virus Res.

[47] Bai F, Wang T, Pal U, Bao F, Gould LH (2005). Use of RNA interference to prevent lethal murine west nile virus infection.. J Infect Dis.

[48] Wiebusch L, Truss M, Hagemeier C (2004). Inhibition of human cytomegalovirus replication by small interfering RNAs.. J Gen Virol.

[49] Ge Q, Eisen HN, Chen J (2004). Use of siRNAs to prevent and treat influenza virus infection.. Virus Res.

[50] Alhoot MA, Wang SM, Sekaran SD (2011). Inhibition of Dengue Virus Entry and Multiplication into Monocytes Using RNA Interference.. PLoS Negl Trop Dis.

[51] Ang F, Wong AP, Ng MM, Chu JJ (2010). Small interference RNA profiling reveals the essential role of human membrane trafficking genes in mediating the infectious entry of dengue virus.. Virology journal.

[52] Padwad YS, Mishra KP, Jain M, Chanda S, Karan D (2009). RNA interference mediated silencing of Hsp60 gene in human monocytic myeloma cell line U937 revealed decreased dengue virus multiplication.. Immunobiology.

[53] Seneviratne SL, Malavige GN, de Silva HJ (2006). Pathogenesis of liver involvement during dengue viral infections.. Trans R Soc Trop Med Hyg.

[54] Couvelard A, Marianneau P, Bedel C, Drouet MT, Vachon F (1999). Report of a fatal case of dengue infection with hepatitis: demonstration of dengue antigens in hepatocytes and liver apoptosis.. Hum Pathol.

[55] Kuo CH, Tai DI, Chang-Chien CS, Lan CK, Chiou SS (1992). Liver biochemical tests and dengue fever.. Am J Trop Med Hyg.

[56] Das S, Garver L, Ramirez JR, Xi Z, Dimopoulos G (2007). Protocol for Dengue Infections in Mosquitoes (A. aegypti) and Infection Phenotype Determination.. J Vis Exp.

[57] De Madrid AT, Porterfield JS (1969). A simple micro-culture method for the study of group B arboviruses.. Bulletin of the World Health Organization.

[58] Duxbury MS, Whang EE (2004). RNA interference: a practical approach.. The Journal of surgical research.

[59] Jackson AL, Burchard J, Leake D, Reynolds A, Schelter J (2006). Position-specific chemical modification of siRNAs reduces “off-target” transcript silencing.. RNA.

[60] de Jonge HJ, Fehrmann RS, de Bont ES, Hofstra RM, Gerbens F (2007). Evidence based selection of housekeeping genes.. PLoS One.

[61] Vandesompele J, De Preter K, Pattyn F, Poppe B, Van Roy N (2002). Accurate normalization of real-time quantitative RT-PCR data by geometric averaging of multiple internal control genes.. Genome Biol.

[62] Lambeth CR, White LJ, Johnston RE, de Silva AM (2005). Flow cytometry-based assay for titrating dengue virus.. Journal of clinical microbiology.

[63] Wang WK, Chao DY, Kao CL, Wu HC, Liu YC (2003). High levels of plasma dengue viral load during defervescence in patients with dengue hemorrhagic fever: implications for pathogenesis.. Virology.

[64] Altmeyer R (2004). Virus attachment and entry offer numerous targets for antiviral therapy.. Curr Pharm Des.

[65] Acosta EG, Talarico LB, Damonte EB (2008). Cell entry of dengue virus.. Future Virology.

[66] Dey A, Kessova IG, Cederbaum AI (2006). Decreased protein and mRNA expression of ER stress proteins GRP78 and GRP94 in HepG2 cells over-expressing CYP2E1.. Arch Biochem Biophys.

[67] Sugawara S, Takeda K, Lee A, Dennert G (1993). Suppression of stress protein GRP78 induction in tumor B/C10ME eliminates resistance to cell mediated cytotoxicity.. Cancer Res.

[68] Hendershot LM, Ting J, Lee AS (1988). Identity of the immunoglobulin heavy-chain-binding protein with the 78,000-dalton glucose-regulated protein and the role of posttranslational modifications in its binding function.. Mol Cell Biol.

[69] Lee AS (2005). The ER chaperone and signaling regulator GRP78/BiP as a monitor of endoplasmic reticulum stress.. Methods.

[70] Dong D, Ko B, Baumeister P, Swenson S, Costa F (2005). Vascular targeting and antiangiogenesis agents induce drug resistance effector GRP78 within the tumor microenvironment.. Cancer Res.

[71] Satoh M, Nakai A, Sokawa Y, Hirayoshi K, Nagata K (1993). Modulation of the phosphorylation of glucose-regulated protein, GRP78, by transformation and inhibition of glycosylation.. Exp Cell Res.

[72] Neumann-Staubitz P, Hall SL, Kuo J, Jackson AP (2010). Characterization of a temperature-sensitive vertebrate clathrin heavy chain mutant as a tool to study clathrin-dependent events in vivo.. PLoS One.

[73] Acton SL, Brodsky FM (1990). Predominance of clathrin light chain LCb correlates with the presence of a regulated secretory pathway.. J Cell Biol.

[74] Hinrichsen L, Harborth J, Andrees L, Weber K, Ungewickell EJ (2003). Effect of clathrin heavy chain- and alpha-adaptin-specific small inhibitory RNAs on endocytic accessory proteins and receptor trafficking in HeLa cells.. J Biol Chem.

[75] Sandoval P, Saeed F, Pisitkun T, Knepper M (2011). Protein Half Lives in mpkCCD Epithelial Cells (Single-Point Method).. http://helixweb.nih.gov/ESBL/Database/ProteinHalfLives/index.html.

[76] Chavez-Salinas S, Ceballos-Olvera I, Reyes-Del Valle J, Medina F, Del Angel RM (2008). Heat shock effect upon dengue virus replication into U937 cells.. Virus Res.

[77] Zhang K, Kaufman RJ (2004). Signaling the unfolded protein response from the endoplasmic reticulum.. Journal of Biological Chemistry.

[78] Klomporn P, Panyasrivanit M, Wikan N, Smith DR (2011). Dengue infection of monocytic cells activates ER stress pathways, but apoptosis is induced through both extrinsic and intrinsic pathways.. Virology.

[79] Paradkar P, Ooi E, Hanson B, Gubler D, Vasudevan S (2011). Unfolded protein response (UPR) gene expression during antibody-dependent enhanced infection of cultured monocytes correlates with dengue disease severity.. Bioscience Reports.

[80] Ron D, Walter P (2007). Signal integration in the endoplasmic reticulum unfolded protein response.. Nature Reviews Molecular Cell Biology.

[81] Umareddy I, Pluquet O, Wang QY, Vasudevan SG, Chevet E (2007). Dengue virus serotype infection specifies the activation of the unfolded protein response.. Virol J.

[82] Yu CY, Hsu YW, Liao CL, Lin YL (2006). Flavivirus infection activates the XBP1 pathway of the unfolded protein response to cope with endoplasmic reticulum stress.. J Virol.

[83] Hurtley SM, Bole DG, Hoover-Litty H, Helenius A, Copeland CS (1989). Interactions of misfolded influenza virus hemagglutinin with binding protein (BiP).. The Journal of cell biology.

[84] Wati S, Soo ML, Zilm P, Li P, Paton AW (2009). Dengue virus infection induces upregulation of GRP78, which acts to chaperone viral antigen production.. Journal of virology.

[85] Limjindaporn T, Wongwiwat W, Noisakran S, Srisawat C, Netsawang J (2009). Interaction of dengue virus envelope protein with endoplasmic reticulum-resident chaperones facilitates dengue virus production.. Biochemical and Biophysical Research Communications.

[86] Wang M, Ye R, Barron E, Baumeister P, Mao C (2009). Essential role of the unfolded protein response regulator GRP78/BiP in protection from neuronal apoptosis.. Cell Death & Differentiation.

[87] Fujita Y, Krause G, Scheffner M, Zechner D, Leddy HE (2002). Hakai, a c-Cbl-like protein, ubiquitinates and induces endocytosis of the E-cadherin complex.. Nat Cell Biol.

[88] Krishnan MN, Ng A, Sukumaran B, Gilfoy FD, Uchil PD (2008). RNA interference screen for human genes associated with West Nile virus infection.. Nature.

[89] Stevenson M (2004). Therapeutic potential of RNA interference.. N Engl J Med.

[90] Wang X-L, Nguyen T, Gillespie D, Jensen R, Lu Z-R (2008). A multifunctional and reversibly polymerizable carrier for efficient siRNA delivery.. Biomaterials.

[91] Subramanya S, Kim SS, Abraham S, Yao J, Kumar M (2010). Targeted delivery of small interfering RNA to human dendritic cells to suppress dengue virus infection and associated proinflammatory cytokine production.. J Virol.

